# Implementation of a new emergency medical communication centre organization in Finland - an evaluation, with performance indicators

**DOI:** 10.1186/1757-7241-19-19

**Published:** 2011-03-31

**Authors:** Veronica Lindström, Jukka Pappinen, Ann-Charlotte Falk, Maaret Castrén

**Affiliations:** 1 Karolinska Institutet, Department of Clinical Science and Education and Section of Emergency Medicine Södersjukhuset, Södersjukhuset, Stockholm, Sweden; 2 Finn HEMS, Lentäjäntie, Vantaa, Finland; 3 Karolinska Institutet, Department of Neurobiology, Care Sciences and Society, Stockholm, Sweden; 4 Karolinska Institutet, Department of Clinical Science and Education and Section of Emergency Medicine Södersjukhuset, Stockholm, Sweden

## Abstract

**Background:**

There is a great variety in how emergency medical communication centers (EMCC) are organized in different countries and sometimes, even within countries. Organizational changes in the EMCC have often occurred because of outside world changes, limited resources and the need to control costs, but historically there is often a lack of structured evaluation of these organization changes. The aim of this study was to evaluate if the performance in emergency medical dispatching changed in a smaller community outside Helsinki after the emergency medical call centre organization reform in Finland.

**Methods:**

A retrospective observational study was conducted in the EMCC in southern Finland. The data from the former system, which had municipality-based centers, covered the years 2002-2005 and was collected from several databases. From the new EMCC, data was collected from January 1 to May 31, 2006. Identified performance indicators were used to evaluate and compare the old and new EMCC organizations.

**Results:**

A total of 67 610 emergency calls were analyzed. Of these, 54 026 were from the municipality-based centers and 13 584 were from the new EMCC. Compared to the old municipality-based centers the new EMCC dispatched the highest priority to 7.4 percent of the calls compared to 3.6 percent in the old system. The high priority cases not detected by dispatchers increased significantly (p < 0.001) in the new EMCC organization, and the identification rate of unexpected deaths in the dispatched ambulance assignments was not significantly (p = 0.270) lower compared to the old municipality-based center data.

**Conclusion:**

After implementation of a new EMCC organization in Finland the percentage and number of high priority calls increased. There was a trend, but no statistically significant increase in the emergency medical dispatchers' ability to detect patients with life-threatening conditions despite structured education, regular evaluation and standardization of protocols in the new EMCC organization.

## Background

The emergency medical communication centre (EMCC) and the emergency medical dispatchers (EMD) is a part of the emergency medical services (EMS) and the first link in the chain of survival [1]. There is a great variety in how an EMCC is organized in different countries and sometimes, even within countries [2,3]. In addition there have been major changes in EMCC organizations during the last few years. The changes have often started due to the input of external factors, i.e. limited resources; need to control costs, and discussions concerning management responsibilities [2,3]. However, the assessment of the outcome of the money spent to finance the EMS is generally not evidence-based [4]. A lack of structured evaluations of organizational changes in the EMCC is evident. The aim of an EMCC is still to answer emergency calls immediately, to identify callers' needs and to dispatch the necessary resources wherever and whenever an emergency need occurs. In 2006, the Finnish government implemented a new nationwide EMCC organization with identical conditions, regardless of the EMCC location. The purpose of the organizational changes was to improve the structure of emergency dispatching. The public media and local EMS organizations discussed whether the new EMCC organization was worse for the patient and they argued that there was a risk that patients would not get an ambulance when needed. A recently published study by Määttä and colleagues describes that the EMCC organization reform in Finland had negative effects on the appropriate use of ambulances, and the reform caused prolongation in the answering and processing times of emergency calls in Helsinki, the capital of Finland [5].

### EMCC organization and EMD in Finland - before and now

There used to be 45 municipality-based centers taking emergency calls in Finland. There were no official criteria for how these centers should be organized and all of these municipality-based centers had different ways of dealing with the daily work. The local rescue departments were responsible for each local municipality-based center. The computer systems, data format and evaluation strategies varied from centre to centre. There was no consensus concerning training, education, or competence of the personnel answering the emergency calls in the old municipality-based centers. In 2006, when the nationwide EMCC organization was implemented, the Health Care Services became responsible for the 15 new EMCC. One of the first actions of the new organization was to make the same stipulations regarding the competence and education of the personnel. In the new EMCC organization the EMD needed one and a half years of formalized training to be qualified as a dispatcher.

Since the1980s there have been four dispatching codes (A-D) relating to patients' acuity. The priority codes in the municipality-based centers were not based on legislation but more on common practice in the local organization. During the reform of EMCC organization the priority codes remained the same but became standardized and were regularly monitored. The definitions of prioritizing in the new EMCC organization were:

*Priority code A; *the patient has a life-threatening situation or has been exposed to a high-energy accident. The emergency call should be responded to immediately. The nearest physician unit and ambulance should be dispatched to the scene.

*Priority code B*; there is suspicion of failure of vital functions. The emergency call should be responded to immediately and the nearest ambulance should be dispatched to the scene immediately.

*Priority code C*; the patient needs assessment by an emergency care team. The ambulance must arrive at the scene within 30 minutes.

*Priority code D*; no suspicion of failure of vital functions. The patient can wait, the ambulance must arrive at the scene within 120 minutes [6].

To support the EMD assessment, both the municipality-based centers and the new EMCC used an assessment guide book with 57 medical prioritizing criteria for chief complaints. These criteria for chief complaints remained the same during the EMCC organization reform but became standardized after the organizational change [7]. The dispatching codes consisting of priority and chief complaint were used in the feedback system utilized by ambulances to send feedback to the EMCC concerning the patient's chief complaint and acuity when ambulance personnel arrived at the scene [7]. If the patient was not transported, the ambulances sent feedback to the EMD with a code explaining the reason for not transporting the patient to the hospital. The ambulances have a nine-point classification system regarding non-transport to hospital [6] The feedback system was used in the municipality-based centers but was not regularly monitored and standardized as in the new EMCC organization.

The aim of this study was to evaluate if the performance in emergency medical dispatching changed in a smaller community outside Helsinki after the emergency medical call centre organization reform in Finland.

## Material and methods

A retrospective observational study was conducted in the EMCC in East and Central Uusimaa, an area of southern Finland where the EMCC covers about 300 000 inhabitants. We identified performance indicators and compared them with data collected before and after the new EMCC organization. The study was approved by the institutional review board.

### Data in this study

The selected old municipality-based centers had computer-based statistical data on EMD assignments and ambulance feedback, which made a comparison on a group level between the old and the new system possible. A convenient sample from the municipality-based centers covered the years 2002-2005 and was collected from several databases. Approximately 40% of all emergency calls during the period 2002-2005 were available from the municipality-based databases. The rest of the data could not be gathered since it was impossible to retrieve it from the old databases. The estimated number of emergency calls in the area was 32 600 per year. From the new EMCC, East and Central Uusimaa, which covers the whole area of the closed municipality-based centers, was collected from January 1 to May 31, 2006. During the study period the population in the area increased from 273 000 to 281 000 and the death rates varied from 1809 to 1820 per year [8].

### Performance indicators

The identification and development of the performance indicators was based on two presumptions made by the research group: a large population will generate an equal rate of emergency calls, and if the EMD follows a predefined protocol, it leads to the same assessment of priority with the same kind of emergency call.

Two performance indicators were identified: priority distribution and underestimation of risk to detect life-threatening situations by the EMD, as displayed in table 1.

**Table 1:** Identified performance indicators

Performance indicators	Description
Priority distribution	General indicator of EMCC quality. An emergency call assessment and action should result in similar distribution of priority classes in different EMCC
Underestimation of priority	Life-threatening situations not detected by EMD and thus classified with a lower priority code than actually needed

### Variables

The data from both the municipality-based centers and the new EMCC contained:

- Dispatcher's assessment concerning priority (A-D)

- Underestimation of priority: feedback from ambulance; dispatch assessment C+D compared to ambulance feedback A+B

- The feedback from the ambulance to the dispatcher that the patient was "dead at the scene"

Inter-hospital transports were excluded from both data sets and no individual assignment could be distinguished from the data sets.

### Procedure

The data analysis regarding the performance indicator *"Priority distribution" *was based on the EMD assessment of priority A-D. A comparison on group level between new and old EMCCs was made. The analyses concerning *"Underestimation of priority" *were based on EMD-assessed priority and ambulance feedback to the centers concerning priority code A-D and feedback that the "patient died at the scene". When ambulance feedback to the center was *"patient died at the scene" *and EMD assessment and dispatching was anything other than priority code A-B (immediate response), these assignments were evaluated as non-correctly assessed by EMD.

Descriptive statistical procedures were computed using the PASW version 18.0 program. Categorical variables were compared by means of Pearson's chi-square test. Risk ratio (RR) and 95% confidence intervals (CI) were calculated by logistic regression. Probability that was the same or below 0.01 was accepted as statistically significant.

## Results

A total of 67 610 emergency calls were analyzed, and of these, 54 026 (79.9%) were from the municipality-based centers, and 13 584 (20%) were from the new EMCC.

A comparison between the municipality-based centers and the new EMCC indicates that priority codes A and C were used in a different way in the new system, with more priority A and fewer priority C dispatch assessments as compared to the old system (table 2).

**Table 2:** Priority distribution in the municipality-based centers and the new EMCC

	Municipality-based centers	EMCC
	Total n = 54 026	Total n = 13 584
Dispatch priority	n (%)	n (%)
Priority A	1 973 (3.6)	981 (7.4)
Priority B	14 361 (26.8)	3 603 (27.1)
Priority C	19 144 (35.7)	4 189 (31.6)
Priority D	18 025 (33.6)	4 476 (33.7)

When comparing the new EMCC with the municipality-based centers using the performance indicator, "Underestimation of priority", the municipality-based centers' data showed that in 0.95 percent (n = 506) of cases the ambulance was dispatched as a low-priority assignment (code C & D) and the patient was transported to the hospital with lights and sirens (code A & B). Similar assignments analyzed from the new EMCC showed 183 cases (1.38%). The difference was significant (p < 0.001). The Risk Ratio for underestimation was higher (RR 1.46) for the new EMCC compared to the municipality-based centers.

In relation to the EMD ability to detect patients in life-threatening situations, the municipality-based centers' data showed a total of 520 assignments where the patient died at the scene. Of those cases, 23.5 percent (n = 122) occurred with low-prioritized calls (code C & D). In the new EMCC there were166 assignments when the patient died at the scene, and of those 13.9 percent (n = 23) occurred with low-prioritized calls. The difference was not significantly significant (p = 0.27, CI 0.50- 1.22 and RR 0.78).

## Discussion

This study is one of the few that actually tries to evaluate organizational change in the EMCC. Our results indicate that the EMD in the new EMCC organization is better able to identify patients in a life-threatening situation, even though there is no statistical significance. This result is in concordance with a previous study which showed that a well-trained and functioning EMCC is able to detect high-risk patients who require highest-priority [7]. However Määttä and colleagues conclude that the EMCC organization reform in Finland did not affect the accuracy of assessing potentially life-threatening conditions [5]. The varying results between our studies may be caused by the fact that different variables were used to evaluate the organization changes. The EMD has an essential and important role in the early management of patients, and there are some difficulties in evaluating quality and effectiveness of the EMCC, as described by previous authors [4,9]. Still the overall aim for the EMD, regardless of the EMCC organization, is to identify callers' needs and dispatch the necessary resources. An ideal EMD would triage emergency calls with high sensitivity and high specificity [10,11], without unnecessary over and under triage. Compared to the municipality-based centers, the EMD in the new EMCC organization seems to dispatch more ambulance assignments with priority A and fewer priority C assignments. Based on personal experience in the research group, an explanation for this result could be that in the municipality-based centers the rescue department was responsible for its budget, and a priority A assignment would automatically result in dispatching a physician-manned unit, resulting in increased costs for the rescue department.

However, the result may also indicate an over triage in the new EMCC organization, resulting in increased costs [12]. With limited EMS recourses, over triage can also lead to unavailability of ambulances in some situations [13] and should therefore be evaluated on a regular basis.

Compared to the new EMCC, the municipality-based centers' data contained a lower frequency of low-prioritized assignments where the ambulance transported the patient to hospital using blue lights and sirens. A possible explanation could be that there have been changes in the treatment and priority assessment of certain groups of patients since the transition into the new EMCC organization, for example stroke patients.

Due to the absence of data from the old organization it is difficult to draw any conclusions from the results as to why there are differences between the old and the new organizations. A reasonable conclusion is that the transition from the old to the new EMCC organization was poorly designed and implemented. There was no organized collection of data that could allow for a structured evaluation of the organizational changes. It is evident that a well-planned evaluation of changes in the organizations, before they are actually made, is the only way to determine if a change was beneficial or not. We also need defined performance indicators in order to compare the results rather than just describe them. Clear definitions are also needed to state clearly what over and under triage actually mean. Further investigation of possible performance indicators to compare organizations or protocol changes in the EMCC would be of great interest.

### Limitations

There are some limitations that have to be considered in our study. First, the study was a retrospective study and was not planned before the actual change took place. Another limitation is that there are no internationally defined performance indicators for emergency medical dispatching. The fact that the data from the new EMCC was obtained over a five-month period when the new EMCC organization had only been in operation for a short time may have affected the results. The EMD adaptation to new routines in the organization might not have been secured. Other limitations are that data from the old centers were collected during a four-year period and that changes in the diseases may have occurred over time. This may have had an impact on the results concerning the ability of EMDs to identify patients in life-threatening situations. However, the death rate in the area did not change during the study period [8] and therefore it should not have affected the results. The sample size concerning both pre-hospital deaths and priority A assignments was quite small, and was spread over several years.

Another bias in our result could have been caused by the impact of external factors such as ambulance personnel training, EMD & EMCC management, and sent feedback codes. Data from the Swedish EMCC indicates that eight percent of the feedback sent from the ambulance to EMCC is incorrect [14]. If this were also true in our material this could have had an impact on our result.

Collecting data from multiple EMCCs and/or data over a complete year would have reduced this bias. The municipality-based centers were selected on the basis that there were materials available; this could imply that the selected centers may have been better organized compared to other centers. The effects of the EMCC organization reform may have been clarified if more data from municipality-based centers had been collected and included in this study.

## Conclusion

There was a trend, but no statistically significant increase, in the EMDs' ability to detect patients with life-threatening conditions despite structured education, regular evaluation and standardization of protocols in the new EMCC organization.

## Competing interests

There are no financial competing interests (political, personal, religious, ideological, academic, intellectual, commercial or any other) to declare in relation to this manuscript

## Authors' contributions

JP and MC designed the study. JP collected data. Analyses were made by VL, JP, ACF and MC, VL drafted the manuscript, and all authors contributed substantially to the manuscript. All authors have read and approved the final manuscript.
